# Life threatening pneumonia in a lupus patient: a case report

**DOI:** 10.1186/1757-1626-1-70

**Published:** 2008-07-31

**Authors:** Maciej Kupczyk, Adam Antczak, Piotr Kuna, Paweł Górski

**Affiliations:** 1 Dept of Internal Medicine, Asthma and Allergy, Medical University of Łódź, Łódź, Poland; 2 Dept of Pneumonology and Allergy, Medical University of Łódź, Łódź, Poland

## Abstract

We report a case of systemic lupus erythematosus (SLE) in a 44-year old Caucasian woman complicated with pneumonia and severe respiratory failure requiring ICU treatment and mechanical ventilation. Symptoms developed in a generally well controlled SLE course after sudden stop in immunosupresant therapy (methotrexate, cyclosporin and methylprednisolone). A fulminant course of the disease, an interstitial pattern in a high resolution computed tomography (HRCT) and negative repeated sputum, blood and bronchoaspirate cultures enabled diagnosis of fulminant lupus pneumonitis. The response to pulses of cyclophosphamide and methylprednisolone was good but complicated with a significant leukopenia. HRCT confirmed significant remission of pulmonary changes. Fulminant lupus pneumonitis is a rare but potentially life threatening complication of SLE. Differential diagnosis requires exclusion of pneumonia induced by pathogens such as Pneumocystis jirovevecii (carinii) and Mycobacterium sp. Intensive immunosuppressive therapy and close cooperation between ICU, pulmonology and rheumatology departments is necessary in such a case to minimalize the risk of fatal outcome.

## Introduction

Systemic lupus erythematosus (SLE) is an autoimmune chronic systemic disease which can involve several organs such as skin, lungs, brain and heart. Pulmonary manifestations of SLE can include a wide spectrum of diseases such as pleuritis, pneumonia, pulmonary embolism, pneumothorax and pulmonary haemorrhage [1,2]. As the basic treatment of SLE include several drugs inducing immunosuppression pneumonia and acute respiratory distress syndrome (ARDS) followed by sepsis are the most common causes of admission to the ICU and fatal outcome in these patients. Only few cases of non-infectious fulminant lupus pneumonitis mimicking, by its interstitial pattern, atypical pneumonia has been presented in literature to date. Differential diagnosis and treatment of this condition represent a real challenge but only early introduction of intensive immunosuppressive treatment and close cooperation between ICU, pulmonology and rheumatology departments reduce the risk of fatal outcome.

## Case presentation

**Figure 1:**
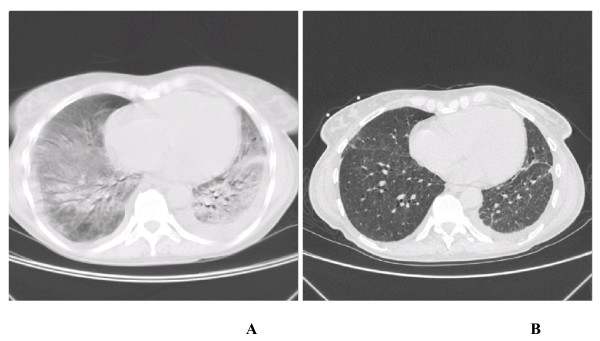
Selected scans from high resolution computed tomography before (A) and after (B) 15 days of treatment.

A 44-year old white woman was admitted to our hospital complaining of dyspnoea, non-productive cough and 40°C fever for the past 2 days. She had been diagnosed with SLE at the age of 18 years. The course of her SLE was well controlled in an outpatient clinic. She had never smoked. On examination on admission she was febrile, with tachycardia (HR 100/min) and tachypnoe 24/min. On auscultation loud crackles were audible over the both lungs. Chest X-ray revealed an interstitial pattern with bilateral ground-glass shadow. Her WBC was 11.4 × 10^3^/μl, C-reactive protein 198 mg/l, sedimentation rate 90 mm after 1 hour. Blood gases measurement in the arterialized blood from the capillary vessels revealed severe respiratory failure with hypoxaemia (pO_2 _39.4 mmHg, pCO_2 _30.5 mmHg, Sat 75.5%). An atypical pneumonia was suspected. Intravenous antibiotics (ciprofloxacin and spiramicin), oxygen (2 l/min) and steroids (methylprednisolone in the dose 1 mg/1 kg of the body mass orally) were started. Repeated blood gases evaluations showed no improvement thus the rate of oxygen flow was increased to 4 l/min and methylprednisolone to 0.5 g daily intravenously. After 2 days of such treatment a significant improvement was observed. She was afebrile, with HR 70/min, respiratory rate 16/min, pO_2 _64.0 mmHg, pCO_2 _28.4 mmHg, Sat 93.1%. On the 3^rd ^day after admittion patient's condition suddenly deteriorated with severe dyspnoe, fever (39°C), shivers, HR 130 – 150/min and respiratory rate 45/min. She has been transferred to the ICU, required endotracheal intubation and mechanical ventilation. High resolution computed tomography (HRCT) showed ground glass opacity (Figure. 1A.). There were negative repeated sputum and blood cultures. Bronchoalveolar lavage (BAL) cultures were also negative.

**Figure 2:**
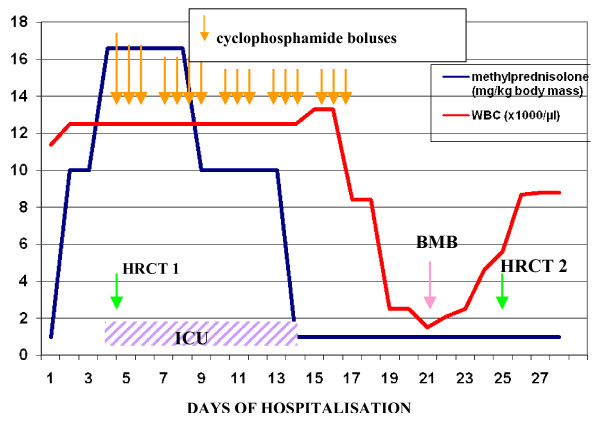
**Scheme of the fulminant lupus pneumonitis patient diagnosis and treatment (WBC – white blood cell count, HRCT – high resolution tomography, ICU-intensive care unit, BMB – bone marrow biopsy)**.

The past medical history included symptoms of respiratory tract infection, arthralgia, oral ulcers, fever and skin rush noted in November 2004. Anti-nuclear antibodies (ANA) level was 1:1280 (range: till 1:80). Patient was diagnosed in an immunology outpatient clinic as a recurrence of SLE and effective treatment with methotrexate, cyclosporin and methylprednisolone was introduced. For an unknown reason the treatment has been suddenly stopped and changed to monotherapy with chloroquine just 4 days before the development of symptoms and admission to our hospital. The level of pANA was 1:2560. Taking this and negative sputum and blood cultures into consideration we diagnosed fulminant lupus pneumonitis. Intensive immunosuppressive treatment has been introduced with pulses of cyclophosphamide (CP) (0.6 g iv/daily on the first day in the ICU, 0.4 g on the 2^nd ^and 3^rd ^days, 0.2 g for the next 4 days and with following 0.1 g iv and later orally), methylprednisolone (1 g iv/daily) (Figure. 2.) and mesna to prevent the urotoxicity of CP. The patient's condition gradually improved and she was extubated on the 5^th ^day and transferred to the Pneumonology Department. A significant leukopenia as a side effect of the immunosuppressive agents was observed (drop in the WBC from 8.4 × 10^3^/μl down to 1.5 × 10^3^/μl during 4 days) (Figure. 2). Despite preventive antibiotic treatment (ceftriaxone 4 g iv/daily) 39°C fever developed. Antibiotics has been changed to levofloxacine (1 g iv daily) and amikacin (0.5 g iv daily) and patient felt better. Doses of cyclophosphamide previously reduced to 100 mg po daily had been withdrawn. Methylprednisolone was given orally 1 mg/kg of the body mass daily. The bone marrow biopsy has been performed showing intensive hematopoietic cells differentiation and maturation which has been mirrored in the peripheral blood count (increase in the WBC to 8.8 × 10^3^/μl after 7 days without granulocyte colony-stimulating factor treatment). She was discharged 1 week later with HRCT confirmed significant remission of pulmonary changes (Figure. 1B.) and referred to follow up in an outpatient clinic.

## Discussion

The above described case presents fulminant lupus pneumonitis a rare but life threatening complication of SLE. Pulmonary manifestations of SLE can include a wide spectrum of diseases such as pleuritis, pneumonia, pulmonary embolism, pneumothorax and pulmonary haemorrhage [1,2]. Hsu et al in the group of 51 critically ill patients with SLE treated in the ICU found the mortality rate about 47% with pneumonia and acute respiratory distress syndrome (ARDS) followed by sepsis as the most common cause of admission [3]. The pathogens cultured in studied cases included Pseudomonas aeruginosa, Salmonella sp, Staphylococcus aureus and epidermidis, Streptococcus pneumoniae, E. coli and Acinetobacter baumannii. In two patients disseminated tuberculosis was diagnosed. These findings are not surprising if we remember that glucocorticosteroids and other drugs used in the treatment of SLE induce significant immunosupression thus increasing the risk of all kinds of infections. In line with the paper of Hsu et al septic shock is associated with higher risk of fatal outcome in SLE patient treated in ICU, that is why identification of pathogen and immediate antimicrobial therapy is of great importance [3]. Only one patient (1,6%) from the study group [3] has been diagnosed with noninfectious pneumonitis. Comer et al reported another case of a patient with SLE, whose pregnancy was complicated by fulminant pneumonitis and pericarditis [4]. Single cases has been also presented by other authors [5,6]. Isbister et al described a 14 year old girl with SLE, complicated with lupus pneumonitis, acute renal failure and aplasia [5]. Plasmapheresis, dialysis and immunosuppressive therapy were useful in the treatment. Mok CC et al described two clinically very similar cases [6]. One patient was confirmed to have coronavirus pneumonia while the other had fulminant lupus pneumonitis.

Diagnosis of fulminant lupus pneumonitis is a real challenge. As presented above several patogens should be taken into consideration in a case of interstitial pneumonitis including but not limited to viruses [7], Pneumocystis carinii [8] and Mycobacterium sp [9]. In the case we present diagnosis has been made basing on a data from several negative cultures and striking history of sudden reduction, not increase in the dosis of the immunosuppressive agents. Intensive immunosuppressive treatment including glucocorticosteroids, cyclophosphamide, methotrexate, cyclosporin and in selected cases plasmapheresis should be introduced. A close cooperation between ICU, pulmonology and rheumatology departments is required in such a case to minimalize the risk of fatal outcome.

## List of abbreviations

ANA: Anti-nuclear antibodies; ARDS: Acute respiratory distress syndrome; BAL: Bronchoalveolar lavage; BMB: Bone marrow biopsy; CP: Cyclophosphamide; HR: Heart rate; HRCT: High resolution computed tomography; ICU: Intensive care unit; min: Minutes; μl: micro liter; pCO_2:_ Partial presurre of carbon dioxide; pO_2:_ Partial pressure of oxygen; Sat: Saturation; SLE: Systemic lupus erythematosus; sp.: Species; WBC: white blood count.

## Competing interests

The authors declare that they have no competing interests.

## Authors' contributions

MK analyzed and interpreted the patient data regarding the pulmonary disease and was a major contributor in writing the manuscript, AA analyzed and interpreted the patient data regarding the pulmonary disease, performed bronchoscopies and BAL, PK analyzed and interpreted the patient data regarding the rheumatological disease and was a contributor in writing the manuscript, PG analyzed and interpreted the patient data regarding the rheumatological disease and results of HRCT. All authors read and aprooved the final manuscript.

## Consent

Written informed consent was obtained from the patient for publication of this case report and accompanying images. A copy of the written consent is available for review by the Editor-in-Chief of this journal.

## References

[B1] Beresford MW, Cleary AG, Sills JA, Couriel J, Davidson JE (2005). Cardio-pulmonary involvement in juvenile systemic lupus erythematosus. Lupus.

[B2] Ciftçi E, Yalçinkaya F, Ince E, Ekim M, Ileri M, Orgerin Z, Fitöz S, Güriz H, Aysev AD, Dogru U (2004). Pulmonary involvement in childhood-onset systemic lupus erythematosus: a report of five cases. Rheumatology.

[B3] Hsu CL, Chen KY, Yeh PS, Hsu YL, Chang HT, Shau WY, Yu CL, Yang PC (2005). Outcome and prognostic factors in critically ill patients with systemic lupus erythematosus: a retrospective study. Critical Care.

[B4] Comer M, D'Cruz D, Thompson I, Erskine K, Dacre J (1996). Pneumonitis in a lupus twin pregnancy: a case report. Lupus.

[B5] Isbister JP, Ralston M, Hayes JM, Wright R (1981). Fulminant lupus pneumonitis with acute renal failure and RBC aplasia. Successful management with plasmapheresis and immunosuppression. Arch Intern Med.

[B6] Mok CC, Ying KY (2004). Lupus pneumonitis or severe acute respiratory syndrome?. Lupus.

[B7] Tokunaga Y, Takenaka K, Asayama R, Shibuya T (1996). Cytomegalovirus-induced interstitial pneumonitis in a patient with systemic lupus erythematosus. Intern Med.

[B8] Kadoya A, Okada J, Iikuni Y, Kondo H (1996). Risk factors for Pneumocystis carinii pneumonia in patients with polymyositis/dermatomyositis or systemic lupus erythematosus. J Rheumatol.

[B9] Huang YC, Lin YT, Yang YH, Wang SJ, Yang CM, Chiang BL (2001). Acute lupus pneumonitis mimicking pulmonary tuberculosis: a case report. J Microbiol Immunol Infect.

